# Necrosis of the Alveolar Edge Caused by Tincture of Iodine

**Published:** 1912-09-15

**Authors:** 

**Affiliations:** Toulouse


### NECROSIS OF THE ALVEOLAR EDGE CAUSED BY TINCTURE OF IODINE.

BY DR. NUX, TOULOUSE.

A soldier, troubled with a large dental abscess, had a second right upper molar extracted. Several days after the removal of the tooth, thinking that healing was not taking place, he went to a dentist who advised painting the gum with tincture of iodine. The patient carried out the instructions with the conscience of one accustomed to obey orders, and so well did he do it that several days later the alveolar border was laid bare and the wound had no tendency to heal. Not being able to perform his duties under the circumstances, he came to the Military Hospital to have this wound, which took so long to heal, cared for.

After several days' treatment, and the usual antiseptic mouth washes having been tried, the wound made no progress. In the absence of a well-organized dental service at the hospital, I was asked to see the patient and give my opinion on this case, which was a little out of the ordinary surgical routine. Upon examining the patient it was at once seen that a large area of the alveolar border was absolutely bare, and reached from the second upper right premolar to the third molar. The bone was therefore uncovered for about 2 centimetres in length and half centimetre deep, and the same breadth.

The alveolar crest was thick and eburnated, and the gum moreover was in good condition, without hypertrophy, though smooth and polished, and ended abruptly at the edge of the bone.

The patient then mentioned the numerous paintings he had made with iodine, two or three a day, and it was easy to see the result of this.

As regards the treatment to be followed, it was not a question of a plastic operation, because this bone, denuded for so long, had only kept its good appearance on account of the antiseptic, but at the same time caustic washings, with tincture of iodine, and it would not have been wise to endeavor to enclose it with strips of tissue dissected from the gum and palate and sutured in place. A spontaneous cure could not be counted upon, for the bone left uncovered could not heal until it was covered with soft tissue.

In these circumstances the simplest solution seemed to be to resect the alveolar border a little below the gum and to leave cicatrization to take place of its own accord. This is the treatment that was adopted, and a few days later I had the pleasure of seeing the patient with his gum completely healed. The loss of the alveolar tissue was scarcely greater than that seen in persons who have lost their molars five or six years previously.

This case is interesting to report, on account of its comparative rarity, and also interesting to practitioners who will see that a drug, apparently so inoffensive as tincture of iodine is known to be that it is frequently recommended, may sometimes produce troublesome sequences when it is used by inexperienced persons, who are able to abuse it, in the laudable desire to obtain a more rapid cure.—*Le Laboratoire et Le Progres Dentaire.*

